# Chaperone co-inducer BGP-15 inhibits histone deacetylases and enhances the heat shock response through increased chromatin accessibility

**DOI:** 10.1007/s12192-017-0798-5

**Published:** 2017-05-04

**Authors:** Marek A. Budzyński, Tim Crul, Samu V. Himanen, Noemi Toth, Ferenc Otvos, Lea Sistonen, Laszlo Vigh

**Affiliations:** 1 Faculty of Science and Engineering, Cell Biology, Åbo Akademi University, FI-20520 Turku, Finland; 2 Turku Centre for Biotechnology, Åbo Akademi University and University of Turku, FI-20520 Turku, Finland; 3 Biological Research Centre of the Hungarian Academy of Sciences, Szeged, 6726 Hungary

**Keywords:** Heat shock factor protein 1 (HSF1), Histone deacetylase (HDAC), Stress response, Transcription, TSA, VPA

## Abstract

Defects in cellular protein homeostasis are associated with many severe and prevalent pathological conditions such as neurodegenerative diseases, muscle dystrophies, and metabolic disorders. One way to counteract these defects is to improve the protein homeostasis capacity through induction of the heat shock response. Despite numerous attempts to develop strategies for chemical activation of the heat shock response by heat shock transcription factor 1 (HSF1), the underlying mechanisms of drug candidates’ mode of action are poorly understood. To lower the threshold for the heat shock response activation, we used the chaperone co-inducer BGP-15 that was previously shown to have beneficial effects on several proteinopathic disease models. We found that BGP-15 treatment combined with heat stress caused a substantial increase in HSF1-dependent heat shock protein 70 (HSPA1A/B) expression already at a febrile range of temperatures. Moreover, BGP-15 alone inhibited the activity of histone deacetylases (HDACs), thereby increasing chromatin accessibility at multiple genomic loci including the stress-inducible HSPA1A. Intriguingly, treatment with well-known potent HDAC inhibitors trichostatin A and valproic acid enhanced the heat shock response and improved cytoprotection. These results present a new pharmacological strategy for restoring protein homeostasis by inhibiting HDACs, increasing chromatin accessibility, and lowering the threshold for heat shock response activation.

**Electronic supplementary material:**

The online version of this article (doi:10.1007/s12192-017-0798-5) contains supplementary material, which is available to authorized users.

## Introduction

Proper protein function is critical for all organisms. When exposed to stress, such as changes in ambient temperature, UV radiation, or in many pathologies, organisms trigger survival mechanisms to maintain their protein homeostasis. One such survival mechanism is the heat shock response (HSR) that consists of a complex network of inducible molecular chaperones, including heat shock proteins (Hsps), which are under the transcriptional control of heat shock factor 1 (HSF1) (Budzyński and Sistonen 2017). Under normal conditions, Hsps assist nascent proteins to reach their final conformation, whereas, upon exposure to stress, Hsps bind to misfolded proteins to prevent the formation of aggregates and either facilitate their refolding or direct them to degradation (Hartl et al. 2011). Failure to adequately mount the HSR is central to many severe and prevalent pathological conditions, such as neurodegenerative diseases, muscle dystrophies, and metabolic disorders (Chung et al. 2008; Gehrig et al. 2012; Su and Dai 2016). Considering the immense impact of these diseases in the society, therapeutic strategies to restore the HSR are urgently needed. As genetic approaches are not feasible on a human scale, several pharmacological approaches to directly target the pathway of HSF1-mediated HSR have been tested in various disease models (Neef et al. 2010; Calamini et al. 2011; West et al. 2012). For instance, activation of HSF1 by the HSPC1 (Hsp90) inhibitor 17-(allylamino)geldanamycin (17-AAG) ameliorates cytotoxicity in an Alzheimer’s disease model (Chen et al. 2014), and activation of HSF1 by celastrol was shown to reduce toxicity in a cardiomyopathy model (Sharma et al. 2014). However, most of the currently used pharmacological activators of the HSR have cytotoxic effects which severely hamper the drug development.

The hydroximic acid derivative BGP-15 is a small (350 Da) multi-target molecule, which intercalates into membranes and stabilizes their lipid rafts, reduces the levels of reactive oxygen species by enhancing mitochondrial efficiency, and inhibits both poly(adenosine 5′-diphosphate)-ribose]polymerase 1 (PARP-1) and tumour necrosis factor-α-induced pathways (Chung et al. 2008; Gombos et al. 2011; Henstridge et al. 2014; Gungor et al. 2014; Sumegi et al. 2017; Crul et al. 2013). Multiple studies have shown beneficial effects of BGP-15 on proteinopathic disease models, such as insulin resistance (Henstridge et al. 2014; Literáti-Nagy et al. 2014), atrial fibrillation (Zhang et al. 2011), muscle dystrophy (Gehrig et al. 2012; Kennedy et al. 2016), and ventilation-induced diaphragm dysfunction (VIDD) (Salah et al. 2016). In these cases, the BGP-15-mediated improvement of protein homeostasis and survival was reported to be due to its capacity to co-induce Hsps. For instance, BGP-15 treatment resulted in HSPA1A/B (Hsp70) upregulation in rat and rabbit models of insulin resistance, dystrophic mice, and the VIDD rat model (Henstridge et al. 2014; Literáti-Nagy et al. 2014; Salah et al. 2016), while upregulation of DmHSP23 was observed in the *Drosophila* tachycardia model (Zhang et al. 2011). Based on these findings, the term “membrane lipid therapy” pharmaceuticals was introduced as a molecular base for drug discovery and disease treatment through the modulation of cell membrane composition and structure using BGP-15 and other hydroximic acid derivatives (Escribá et al. 2015).

During the activation-attenuation cycle, HSF1 is extensively post-translationally modified, binds to DNA, activates gene transcription, and is subsequently released from its target sites (Hietakangas et al. 2003; Westerheide et al. 2009; Budzyński and Sistonen 2017; Raychaudhuri et al. 2014; Budzyński et al. 2015). Previously, it was reported that the hydroxylamine derivative bimoclomol enhances the expression of Hsps and has a cytoprotective effect upon several stresses including ischaemia (Vígh et al. 1997). Mechanistically, bimoclomol has been shown to bind to HSF1, thereby prolonging HSF1 DNA-binding activity (Hargitai et al. 2003). In this study, we investigated whether the chaperone co-inducing capacity of BGP-15, similarly to bimoclomol, stems from changes in the activation-attenuation cycle of HSF1. We found, however, that BGP-15 accelerates the activation and attenuation of HSF1 upon stress, sensitizes HSF1 by lowering its activation threshold and facilitating Hsp expression at a febrile range of temperatures. Surprisingly, BGP-15 alone inhibits the activity of histone deacetylases (HDACs), resulting in increased chromatin accessibility at multiple genomic loci, including *HSPA1A*. Using well-known potent HDAC inhibitors (trichostatin A and valproic acid), we demonstrate that HDAC inhibition enhances the HSR and provides cytoprotection against proteotoxic insults. Taken together, we propose a new strategy for chemical activation of HSF1, by increasing chromatin accessibility through HDAC inhibition, which subsequently sensitizes and accelerates HSF1 activation under physiological stress conditions.

## Methods

### Cell culture and treatments

Mouse embryonic fibroblasts (MEFs) were cultured in high-glucose Dulbecco’s modified Eagle’s medium containing 10% foetal calf serum, 2 mM l-glutamine, streptomycin (100 g/ml), penicillin (100 U/ml), and 1× MEM non-essential amino acid solution. Heat shock treatments were conducted in a water bath at 40, 42, and 45 °C (for details, see figure legends). BGP-15 was purchased from N-Gene. Trichostatin A (TSA) was dissolved in DMSO, and valproic acid (VPA) and nicotinamide (NAM) were dissolved in water.

### Quantitative RT-PCR (qRT-PCR)

Immediately after treatment, RNA was isolated with RNeasy Mini Kit (Qiagen) according to the manufacturer’s instructions and quantified using a NanoDrop ND-1000 spectrophotometer (Thermo Scientific). One microgram of total RNA was reverse transcribed with the iScript kit (Bio-Rad). KAPA PROBE FAST ABI Prism qPCR Kit (Kapa Biosystems) and SensiFAST SYBR Hi-ROX (Bioline Reagents) were used for qRT-PCRs that were performed with StepOnePlus or QuantStudio 12K Flex Real-Time PCR System. List of the primers and probes used for qRT-PCR is in Supplementary Table S1. Relative quantities of the target gene messenger RNAs (mRNAs) were normalized against their respective 18S RNA (*RNA18S5*). All reactions were run in triplicate from samples derived from at least four biological replicates.

### Chromatin immunoprecipitation (ChIP)

A total of 3 × 10^7^ MEFs were cross-linked immediately after treatment on ice for 10 min with a final concentration of 1% formaldehyde, followed by 5 min quenching in 125 mM glycine. After lysis in Joost’s lysis buffer (1% SDS, 10 mM EDTA, 50 mM Tris-HCl [pH 8.1]) supplemented with 1× Pierce Protease Inhibitor (Thermo Scientific), 0.5 mM PMSF, and 0.2 mM sodium orthovanadate, samples were sonicated for 10 min using a Diagenode Bioruptor, and 1 mg of whole-cell extracts was used for each immunoprecipitation. Samples were pre-cleared with 50% slurry protein G-Sepharose beads (GE Healthcare Life Sciences) saturated with BSA and salmon sperm DNA, and immunoprecipitation was performed overnight at 4 °C using antibodies against HSF1 (SPA-901; Enzo Life Sciences). Normal rabbit serum was used as a non-specific antibody control. After washing of the immunocomplexes, the remaining RNA and proteins were digested by using RNase A and proteinase K, respectively. Cross-links were reversed by incubating the samples overnight at 65 °C. DNA was purified with phenol-chloroform. Samples were analysed by qPCR using QuantStudio 12K Flex Real-Time PCR System (Applied Biosystems). List of the primers used for qPCR is in Supplementary Table S2. Values obtained from non-specific antibody control were subtracted from the immunoprecipitation samples, which were then normalized to values obtained for input samples.

### Nuclear fractionation and HDAC activity assay

8 × 10^6^ MEFs were used for HDAC activity assay. Cells were collected in cold PBS, and nuclear fractionation was conducted using the NE-PER Nuclear and Cytoplasmic Extraction Reagents kit (Thermo Fisher Scientific) according to the manufacturer’s protocol. HDAC activity was measured using a commercially available, non-radioactive HDAC activity assay kit (Active Motif), according to the manufacturer’s instructions. Briefly, 40 μg of nuclear lysates were incubated in HDAC assay buffer containing BOC-(Ac)Lys-pNi-troanilide for 60 min at 37 °C. The reaction was stopped by adding the stop solution, and after adding the complete developing solution, the mixture was incubated for another 15 min at room temperature. Absorbance was measured at 405 nm using the Hidex Sense microplate reader.

### In vitro HDAC activity assay

The inhibitory effect of BGP-15 on HDAC 1, 4, 6, and 10 and SIRT1 was determined using full-length recombinant HDACs and a fluorogenic substrate (both from BPS Bioscience) according to the manufacturer’s instructions. Briefly, the fluorogenic substrate was incubated with purified HDACs with various concentrations of BGP-15, TSA (for HDAC 1, 4, 6, and 10), or NAM (for SIRT1) for 30 min at 37 °C. The reaction was stopped by adding the developing solution, and the mixture was incubated for another 15 min at room temperature. Fluorescence intensity was measured with excitation and emission at a wavelength of 355/460 nm using the Hidex Sense microplate reader. Blank values were subtracted from the sample values, and the samples without an inhibitor were set to value 100. All reactions were run in quadruple using two different aliquots of inhibitors.

### Micrococcal nuclease (MNase) assay

MNase assay was modified from a previously described protocol (Elsing et al. 2014). 1.5 × 10^7^ MEFs were cross-linked with 1% formaldehyde by incubating cells for 10 min at 37 °C, after which 125 mM glycine was added for 5 min at 4 °C. Cell pellets were washed and resuspended in TM2 buffer (10 mM Tris [pH 7.5], 2 mM MgCl_2_, 1 mM DTT, 5 mM PMSF, and 1× Pierce Protease Inhibitor). Samples were divided into two aliquots: one was digested with MNase (New England Biolabs), and the other was sonicated for 12 min using a Bioruptor (Diagenode). Samples were incubated with MNase at a final concentration of 6.3 U/μl for 10 min at 37 °C, after which the reaction was stopped by adding 5% SDS and 50 mM EGTA. 0.2 M NaCl was added, and cross-links were reversed by incubating samples at 65 °C overnight. Samples were treated with RNase A (6 μg/ml) and proteinase K (50 μg/ml). DNA was purified with phenol-chloroform. Samples were analysed by qPCR QuantStudio 12K Flex Real-Time PCR System. List of the primers used for qPCR is in Supplementary Table S2. The enrichment of MNase-digested DNA was normalized to sonicated DNA. Values were compared with non-treated MNase resistance for the transcriptional start site (TSS) region of each of the analysed loci which was set to value 1.

### Cell viability assay

MEFs were grown on 96-well white, clear-bottom, tissue culture plates (PerkinElmer) at density 5 × 10^3^ cells per well. Cells were either exposed to heat shock at 42 or 45 °C (for details, see figure legends) and left to recover for 16 h at 37 °C or left untreated. Culture media were aspirated, 1× Calcein AM (Invitrogen) diluted in PBS was added to the cells, and samples were incubated for 30 min at 37 °C. Fluorescence intensity was measured with excitation and emission at a wavelength of 485/535 nm using th Hidex Sense microplate reader. Blank values were subtracted from the sample values, and the cell death of non-treated non-heat-shocked samples was set to value 0. All reactions were run in quadruple.

### Statistical analysis

All statistical analyses were performed using GraphPad Prism 7 software with tests as indicated in the figure legends.

## Results

### BGP-15 accelerates the activation of HSF1

**Fig. 1:**
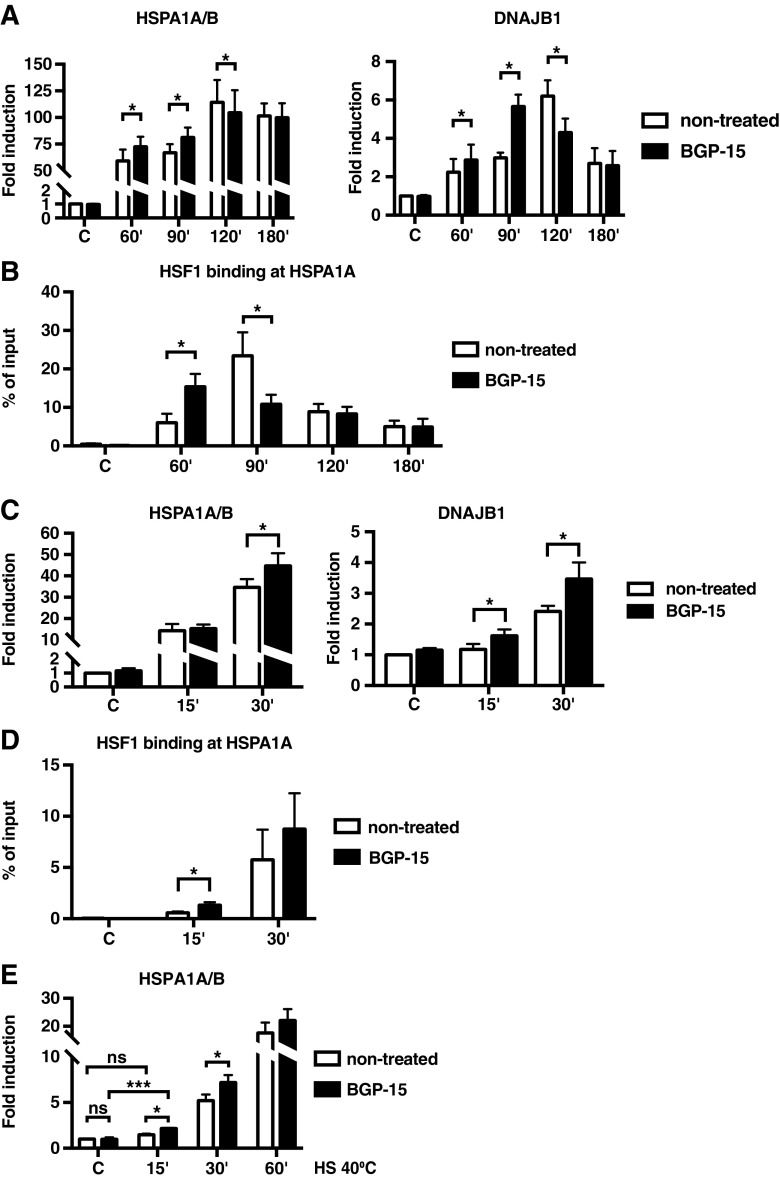
BGP-15 accelerates the activation of HSF1. **a–d** MEFs were treated with or without 10 μM BGP-15 for 1 h, and then exposed to heat shock at 42 °C for indicated times, or left at 37 °C (*C*). **a**, **c** The mRNA levels of *HSPA1A/B* (*Hsp70*) and *DNAJB1* (*Hsp40*) were quantified using qRT-PCR and normalized against *RNA18S5*. The values are shown relative to the respective mRNA levels in the non-treated cells in control conditions (*C*), which was arbitrarily set to value 1. The data are presented as mean values from four independent experiments plus the SEM. **b**, **d** The occupancy of HSF1 at the *HSPA1A* (Hsp70) promoter was analysed by ChIP, followed by qPCR. qPCR values of the immunoprecipitations were normalized to the input values. The data are presented as mean values from four independent experiments plus the SEM. **e** MEFs were treated with or without 10 μM BGP-15 for 1 h, and then exposed to mild heat stress at 40 °C for 15, 30, and 60 min, or left at 37 °C (*C*). The mRNA levels of *HSPA1A/B* (*Hsp70*) were quantified as in **a**. Statistical analyses in **a**–**e** were performed using one-way ANOVA with Holm-Sidak post hoc test. *ns* non-significant. *P ≤ 0.05; ***P ≤ 0.001

To investigate whether BGP-15, similarly to bimoclomol (Hargitai et al. 2003), enhances the HSR through delaying HSF1 attenuation, we treated MEFs with 10 μM BGP-15 for 1 h at 37 °C followed by exposure to 42 °C for 60, 90, 120, and 180 min and analysed the *HSPA1A/B* (*Hsp70*) and *DNAJB1* (*Hsp40*) mRNA levels. During the course of heat exposure, BGP-15 co-induced the expression of *HSPA1A/B* and *DNAJB1* mRNA up to 90 min, resulting in at least 20% increase in the mRNA levels (Fig. 1a). Surprisingly, at 120 min, BGP-15-treated cells exhibited a reduction in the mRNA levels of *HSPA1A/B* and *DNAJB1*, when compared to cells exposed to heat shock alone, indicating that HSF1 might attenuate faster in the presence of BGP-15. However, in cells exposed to heat stress for 180 min, the mRNA levels of Hsp70 and Hsp40 were similar in both BGP-15- and non-treated cells. These observations suggest that, unlike bimoclomol, BGP-15 does not delay the HSF1 attenuation; rather, it enhances Hsp expression in the early phase of HSR.

To verify whether BGP-15 treatment affects HSF1 DNA-binding activity in the context of chromatin, we used chromatin immunoprecipitation assay (ChIP) and compared the occupancy of HSF1 in the presence and absence of BGP-15 at the *HSPA1A* (*Hsp70*) promoter region containing both the proximal and distal heat shock elements (Perry et al. 1994). MEFs were treated as above followed by immunoprecipitation with an HSF1 antibody or normal rabbit serum as a non-specific antibody. Under control conditions, the signal for the occupancy of HSF1 at the *HSPA1A* promoter was hardly detectable in both non-treated and BGP-15-treated cells (Fig. 1b), showing that BGP-15 alone does not induce the DNA-binding activity of HSF1. Upon heat stress, the occupancy of HSF1 increased, and in the BGP-15-treated samples, we observed a 200% higher binding of HSF1 at the 60 min of heat shock when compared to heat shock alone (Fig. 1b), which corresponds to the higher expression levels of Hsps in BGP-15-treated cells (Fig. 1a). However, already at 90 min, HSF1 binding had reduced by 50% in the BGP-15-treated cells when compared to the cells exposed to heat shock alone (Fig. 1b). The HSF1 binding to the *HSPA1A* promoter further decreased at 120 and 180 min of heat stress in both BGP-15- and non-treated cells, indicating HSF1 attenuation from the promoter. These results demonstrate that the potentiating effect of BGP-15 on the HSF1 DNA-binding activity is transient and that BGP-15 accelerates the activation phase of the HSF1 cycle.

To study the HSF1 activation kinetics in more detail, we exposed BGP-15-treated and non-treated MEFs to 42 °C heat shock for 15 and 30 min and analysed the *HSPA1A/B* and *DNAJB1* mRNA levels and HSF1 binding to the *HSPA1A* promoter. As expected, BGP-15 treatment resulted in increased expression of Hsps during the early phase of HSR (Fig. 1c). In addition, the enhanced HSF1 binding to the *HSPA1A* promoter was observed already at 15 min of heat shock (Fig. 1d). These data further confirm that BGP-15 accelerates activation of HSF1 upon proteotoxic stress.

Since in many proteinopathic diseases the HSR is not mounted (Gehrig et al. 2012), we examined whether BGP-15 can activate HSF1-mediated HSR in the context of mild stress. For this purpose, we exposed MEFs to a febrile temperature of 40 °C for 15, 30, and 60 min. This temperature was sufficient to activate the HSR, although the kinetics were delayed and the magnitude of the *HSPA1A/B* induction was lower than at 42 °C (Fig. 1e vs. Fig. 1c). Intriguingly, in the presence of BGP-15, Hsp70 mRNA levels increased by 2.2-fold already at a 15-min heat shock, while in the non-treated cells, there was no change in the levels of *HSPA1A/B* mRNA (Fig. 1e). These results indicate that BGP-15 sensitizes HSF1 by lowering the threshold for its activation.

### BGP-15 inhibits HDAC activity

**Fig. 2:**
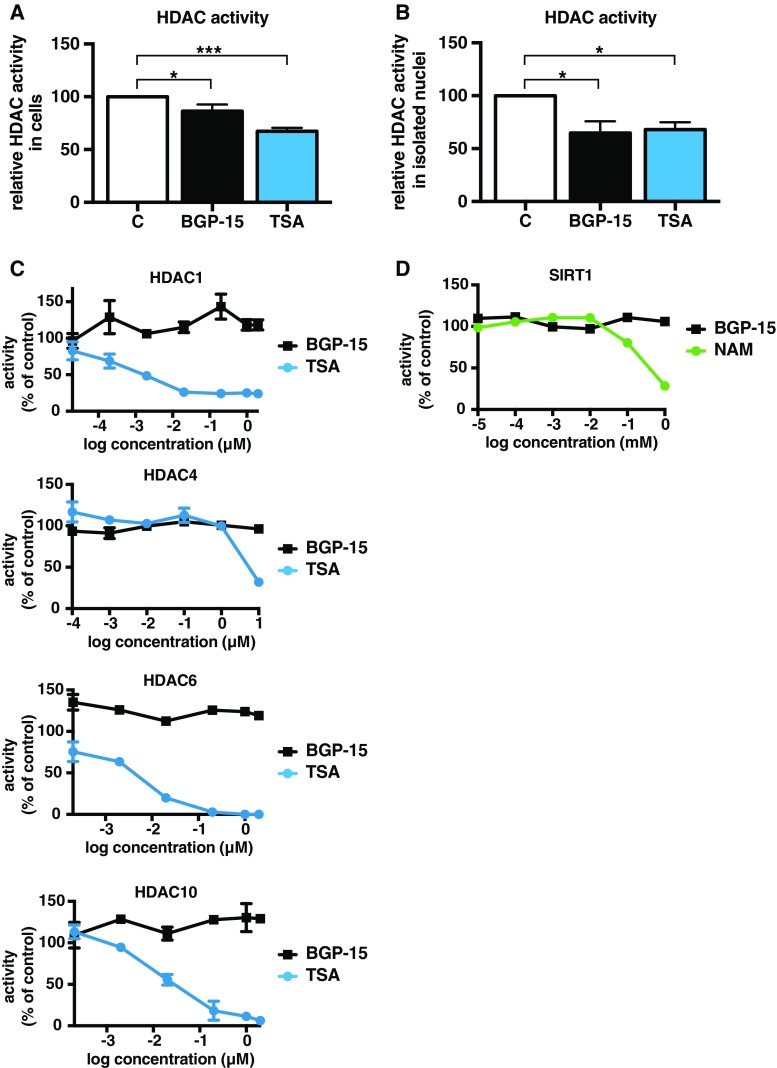
BGP-15 inhibits HDAC activity. HDAC activity upon BGP-15 treatment was assessed both in cells (**a**) and in isolated nuclei (**b**). **a** MEFs were treated with either 10 μM BGP-15 for 1 h, 10 μM trichostatin A (*TSA*) for 4 h, or DMSO as a vehicle control (*C*). Nuclear fractions were isolated and HDAC activity was assessed. HDAC activity in DMSO-treated samples was set to value 100. The data are presented as mean values from at least four independent experiments plus the SEM. **b** Nuclear fractions from MEFs were isolated and treated with either 10 μM BGP-15 or 1 μM TSA or left untreated (*C*), and HDAC activity was assessed. The data are presented as mean values from four independent experiments plus the SEM, where HDAC activity in untreated samples was set to value 100. Statistical analyses for **a** and **b** were performed using one-way ANOVA with Holm-Sidak post hoc test. *P ≤ 0.05; ***P ≤ 0.001. **c** Purified HDAC 1, 4, 6, and 10 were incubated with 1:10 serial dilutions of BGP-15 or TSA for 30 min at 37 °C in the presence of an acetylated fluorogenic substrate. All reactions were run in quadruple. The HDAC activity was quantified as a change in fluorescence intensity of the substrate. HDAC activity in the samples without inhibitors was set to value 100. The data are presented as mean values from two independent experiments ± the SEM. The IC50 values for TSA are 1 nM for HDAC1, 1600 nM for HDAC4, 8 nM for HDAC6, and 14 nM for HDAC10. **d** Purified SIRT1 was incubated with 1:10 serial dilution of BGP-15 or NAM for 30 min at 37 °C in the presence of an acetylated fluorogenic substrate. All reactions were run in quadruple. The SIRT1 activity was quantified as a change in fluoresce intensity of the substrate. SIRT1 activity in the samples without inhibitors was set to value 100. The data are presented as mean values from two independent experiments ± the SEM. The IC50 value for NAM is 108 μM

The mammalian family of histone deacetylases (HDACs) consists of 18 enzymes, of which 11 are zinc-dependent (classes I, II, and IV) and seven require the NAD^+^ co-factor for activity (class III) (Roche and Bertrand 2016). HDACs prevent acetylation of histones and many non-histone proteins including transcription factors. Previously, it has been shown that the activity of HDACs regulates duration and magnitude of HSF1 binding to DNA (Westerheide et al. 2009; Zelin and Freeman 2015). Hence, it is plausible that BGP-15 modulates HSF1 DNA-binding activity through interfering with HDACs. Therefore, we analysed the activity of class I and II HDACs in the presence of BGP-15. First, we treated MEFs with 10 μM BGP-15 for 1 h or 10 μM trichostatin A (TSA) for 4 h. TSA is a well-known class I and II HDAC inhibitor (Yoshidas et al. 1990), which has been shown to effectively inhibit HDACs in MEFs after 2 to 4 h of treatment (Manova et al. 2012). The activity of class I and II HDACs in the nuclear extracts was assessed using a commercially available assay (for details, see “Methods”). As expected, the pre-treatment of cells with TSA resulted in a 30% reduction in the activity of HDACs compared to control cells (Fig. 2a). Intriguingly, cells treated with BGP-15 showed also decreased activity of HDACs (15% reduction).

The activity of HDAC enzymes is regulated through several mechanisms, i.e. signalling pathways and interacting proteins (Sengupta and Seto 2004). Previously, the only direct effect of BGP-15 has been observed on the lipid rafts in the plasma membrane (Gombos et al. 2011). As lipid rafts are nano-scaled domains in the plasma membrane which cluster signalling proteins (Simons and Toomre 2000), BGP-15 might modulate plasma membrane originating signalling cascades which could ultimately also affect HDAC activity. To exclude the possibility that the plasma membrane originating signalling cascades are involved in BGP-15-mediated HDAC inhibition, we performed an HDAC activity assay on isolated nuclei. For this purpose, nuclear extracts from MEFs were incubated with 10 μM BGP-15 or 1 μM TSA for 1 h at 37 °C, and the HDAC activity was measured as above. As shown in Fig. 2b, both BGP-15 and TSA equally inhibited the HDAC activity (35% reduction). These results suggest that BGP-15 inhibits the activity of nuclear HDACs independently of plasma membrane originating signalling cascades.

To gain insight, whether BGP-15 is a direct HDAC inhibitor, we performed an HDAC activity assay with an acetylated fluorogenic substrate and purified HDACs belonging to class I (HDAC1), II (HDAC4, 6, and 10), and III (SIRT1). We did not test HDAC11, a sole member of class IV, since its expression is limited to the muscle, brain, and kidney (Gao et al. 2002). We measured the deacetylation of a substrate in the presence of BGP-15 or the potent HDAC inhibitors TSA or nicotinamide (NAM) (Bitterman et al. 2002). As expected, TSA inhibited the activity of class I and II HDACs (Fig. 2c), while NAM inhibited the activity of SIRT1 (Fig. 2d). However, BGP-15 was unable to inhibit the tested HDACs (Fig. 2c, d), demonstrating that BGP-15 is capable of inhibiting HDACs in cells, but it is not a direct HDAC inhibitor.

### BGP-15 increases chromatin accessibility

**Fig. 3:**
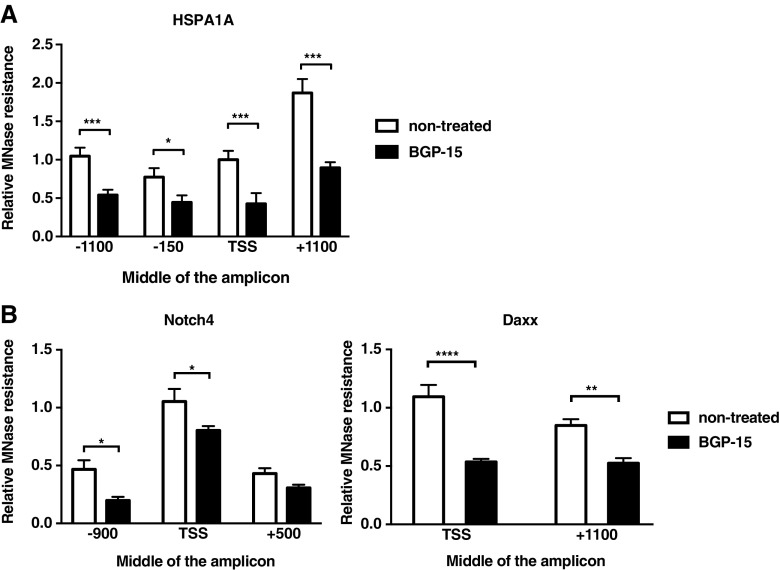
Treatment with BGP-15 results in increased chromatin accessibility. Chromatin structure for genomic region of HSF1 target gene *HSPA1A* (*Hsp70*) (**a**) and two HSR-unrelated genomic regions: *Notch4* (Neurogenic locus notch homologue protein) (Chitnis and Balle-Cuif 2016) and *Daxx* (Death domain associated protein) (Lindsay et al. 2008) (**b**) was analysed using micrococcal nuclease (MNase) sensitivity assay. MEF cells were treated with or without 10 μM BGP-15 for 1 h, and chromatin accessibility was assessed using MNase assay followed by qPCR. The qPCR values obtained from the MNase-treated samples were related to values obtained from sonicated samples and normalized to the non-treated transcriptional start site (TSS) region, which was arbitrarily set to value 1. The data are presented as mean values from four independent experiments plus the SEM. Statistical analysis was performed using multiple *t* tests with Holm-Sidak post hoc test. *P ≤ 0.05; **P ≤ 0.01; ***P ≤ 0.001; ****P < 0.0001

Accessibility of chromatin, including nucleosome assembly and disassembly, is regulated by dynamic acetylation-deacetylation cycles of histones and other chromatin-associated proteins (Henikoff and Shilatifard 2011). HDAC inhibition results in protein hyperacetylation followed by chromatin remodelling and alterations in gene expression (Delcuve et al. 2012). Considering that BGP-15 inhibits the activity of HDACs (Fig. 2), we examined the effect of BGP-15 on the chromatin structure of the promoter and coding region of *HSPA1A*. MEFs were treated with 10 μM BGP-15 for 1 h at 37 °C, and the chromatin structure was analysed using a micrococcal nuclease (MNase) DNA accessibility assay. MNase makes double-stranded cuts between nucleosomes, and higher occupancy of nucleosomes increases the resistance to MNase enzyme activity (Cuatrecasas et al. 1967). Treatment with BGP-15 decreased the MNase resistance throughout the *HSPA1A* promoter and coding region (Fig. 3a), which indicates enhanced chromatin accessibility. Since it is unlikely that the BGP-15-mediated higher accessibility of chromatin would be restricted only to genes involved in the HSR, we performed MNase assay on two HSR-unrelated genomic regions: Notch4 (Neurogenic locus notch homologue protein 4) was chosen as a signalling protein known to affect gene expression (Chitnis and Balle-Cuif 2016), and Daxx (Death domain-associated protein) was selected as an example of general transcriptional regulator (Lindsay et al. 2008). As BGP-15 treatment reduced the MNase resistance of both Notch4 and Daxx genomic loci (Fig. 3b), we conclude that increased chromatin accessibility, in the presence of BGP-15, is not limited to the HSR genes but appears to be a more general phenomenon.

### HDAC inhibition protects cells against proteotoxic stress

**Fig. 4:**
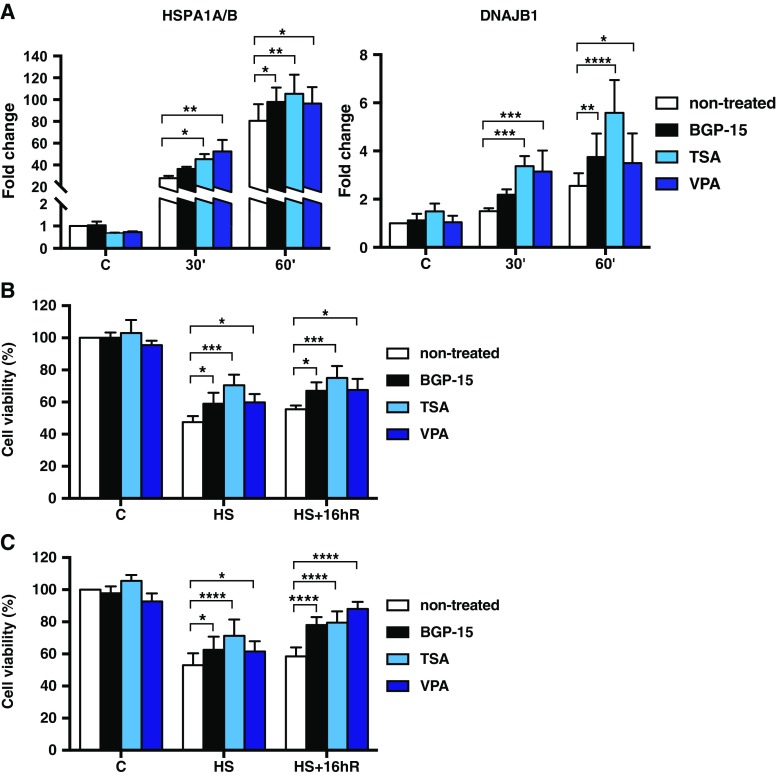
HDAC inhibition protects cells against proteotoxic stress. **a** MEFs were pre-treated with 10 μM BGP-15, 10 μM TSA, 500 μM VPA, or left untreated for 1 h and then exposed to heat shock at 42 °C for indicated times, or left at 37 °C (*C*). The mRNA levels of *HSPA1A/B* (*Hsp70*) and *DNAJB1* (*Hsp40*) were quantified using qRT-PCR and normalized against *RNA18S5*. The values are shown relative to the respective mRNA levels in the non-treated cells under control conditions (*C*), which was arbitrarily set to value 1. The data are presented as mean values from four independent experiments plus the SEM. **b**, **c** MEFs were pre-treated with 10 μM BGP-15, 10 μM TSA, 500 μM VPA, or left untreated for 1 h and then exposed to heat shock (*HS*) for 120 min at 42 °C (**b**) or 30 min at 45 °C (**c**) and left to recover for 16 h at 37 °C (*HS+16hR*). The amount of cell death was measured using Calcein AM dye, and the signal of non-treated, non-heat-shocked cells were set to value 0. The data are presented as mean values from four independent experiments plus the SEM. a–c Statistical analyses were performed using repeated measures two-way ANOVA with Holm-Sidak post hoc test. *P ≤ 0.05; **P ≤ 0.01; ***P ≤ 0.001; ****P < 0.0001

We hypothesized that increased chromatin accessibility by HDAC inhibition enhances the HSR. Thus, we analysed the impact of BGP-15 and two well-known potent HDAC inhibitors, TSA and valproic acid (VPA) (Göttlicher et al. 2002) on Hsp expression. We treated MEFs with either 10 μM BGP-15, 10 μM TSA, or 500 μM VPA for 1 h at 37 °C followed by exposure to 42 °C for 30 and 60 min. As shown in Fig. 4a, the HDAC inhibition did not result in elevated mRNA levels of *HSPA1A/B* or *DNAJB1* under normal conditions. However, upon heat stress, BGP-15, TSA, and VPA exhibited chaperone co-inducing capacity. Elevated expression of Hsps upon HDAC inhibition should yield cytoprotective effect upon stress. To test whether the HDAC inhibition has an impact on cell survival during proteotoxic conditions, we measured cell death in MEFs pre-treated with BGP-15, TSA, or VPA for 1 h and exposed to an acute heat shock at 42 or 45 °C followed by 16 h recovery at 37 °C. Whereas none of the tested HDAC inhibitors had an effect on cell viability under control conditions (Fig. 4b, c), they markedly improved cell survival upon exposure to 120 min of heat shock at 42 °C (Fig. 4b) or 30 min of heat shock at 45 °C (Fig. 4c). Cells treated with HDAC inhibitors and exposed to heat shock at 42 °C were more resistant to stress than the non-treated cells both immediately after heat shock (decrease in cell death by 29% for BGP-15, 52% for TSA, and 33% for VPA) and during the recovery (decrease in cell death by 29% for BGP-15, 41% for TSA, and 25% for VPA). Furthermore, HDAC inhibition offered cytoprotection upon exposure to a severe 30-min heat shock at 45 °C, both immediately after heat shock (decrease in cell death by 29% for BGP-15, 53% for TSA, and 19% for VPA) and during the recovery (decrease in cell death by 37% for BGP-15, 41% for TSA, and 50% for VPA) (Fig. 4c). Taken together, our results revealed that HDAC inhibitors enhance the HSR and improve cell survival upon proteotoxic stress.

## Discussion

Despite numerous attempts to develop strategies for chemical activation of the HSF1 and HSF1-mediated heat shock response (HSR), no effective pharmacological interventions are currently in clinical use. Previous chemical screens focused on the activation of HSF1 in the absence of stress, which in most cases resulted in cytotoxicity. Here, by using BGP-15, we present for the first time the model of enhanced HSR through HDAC inhibition. Treatment with BGP-15 alone inhibits the activity of HDACs, which increases chromatin accessibility for components of the transcription machinery at multiple genomic loci including *HSPA1A*. We demonstrate that BGP-15 accelerates both the activation and attenuation of the HSF1 cycle upon stress. Moreover, BGP-15 sensitizes HSF1 by lowering its activation threshold, thereby enhancing expression of Hsps already at a febrile range of temperatures. We found that similarly to BGP-15, the well-known potent HDAC inhibitors TSA and VPA enhance expression of Hsps, which improves cell survival upon exposure to proteotoxic stress.

BGP-15 is a multi-target drug, with advantageous effects in a variety of proteinopathic disease models (Zhang et al. 2011; Gehrig et al. 2012; Eroglu et al. 2014; Henstridge et al. 2014; Salah et al. 2016). Human clinical trials in healthy individuals (Literáti-Nagy et al. 2009, 2012) and insulin-resistant non-diabetic patients (Literáti-Nagy et al. 2009) demonstrated an excellent safety profile indicating therapeutic potential presumably through Hsp induction. However, BGP-15 not only increases chaperone expression, but it also restores mitochondrial function and inhibits PARP-1 and TNF-α-induced signalling pathways (Chung et al. 2008; Gombos et al. 2011; Henstridge et al. 2014; Gungor et al. 2014; Sumegi et al. 2017). Previously, these effects of BGP-15 were linked to BGP-15-induced remodelling of lipid rafts and fluidization of the membranes (Gombos et al. 2011). In this study, we expand the knowledge of the BGP-15 mode of action by showing that BGP-15 can also interfere with intracellular proteins, such as HDACs, which results in chromatin rearrangement. This novel mode of action of BGP-15 provides an additional explanation for its chaperone co-inducing activity that has been reported in a variety of disease models (Zhang et al. 2011; Gehrig et al. 2012; Eroglu et al. 2014; Henstridge et al. 2014; Salah et al. 2016). Compared with TSA, BGP-15 is less efficient in inhibiting the activity of HDACs in the cellular context, as well as BGP-15 is unable to inhibit the activity of purified HDACs (Fig. 2). The precise mechanism how BGP-15 inhibits HDAC activity is currently unknown. It has been shown that HDACs function as the catalytic core of multi-protein complexes, such as CoREST or the nuclear receptor co-repressor (NCoR) complex (Millard et al. 2017). Within these multi-protein complexes, the HDAC activity is often regulated by the presence of additional HDACs. For example, HDAC4 is essential for the deacetylation activity and transcriptional repression of the NCoR–SMRT–HDAC3 complex (Fischle et al. 2002). As multiple non-HDAC targets have been described for hydroxamate inhibitors (Bantscheff et al. 2011), we speculate that BGP-15 might target non-HDAC subunits and as such could interfere with the formation and activity of the multi-protein HDAC complexes (Millard et al. 2017). Currently, capture compound experiments (Köster et al. 2007), consisting of a biotin-labelled BGP-15, that allow immunoprecipitation of BGP-15 and identification of its interaction partners via mass spectrometry are being pursued in our laboratory.

Being a multi-target drug, it is plausible that the chaperone co-inducing property of BGP-15 consists of combinatorial effects occurring in the plasma membrane and intracellular compartments. Several diseases are described as a network phenomenon in which the partial inhibition of a small number of targets can be more efficient than the complete inhibition of a single target (Csermely et al. 2005; Csermely et al. 2013). Future studies should address the mechanisms by which BGP-15 intervenes with these processes.

**Fig. 5:**
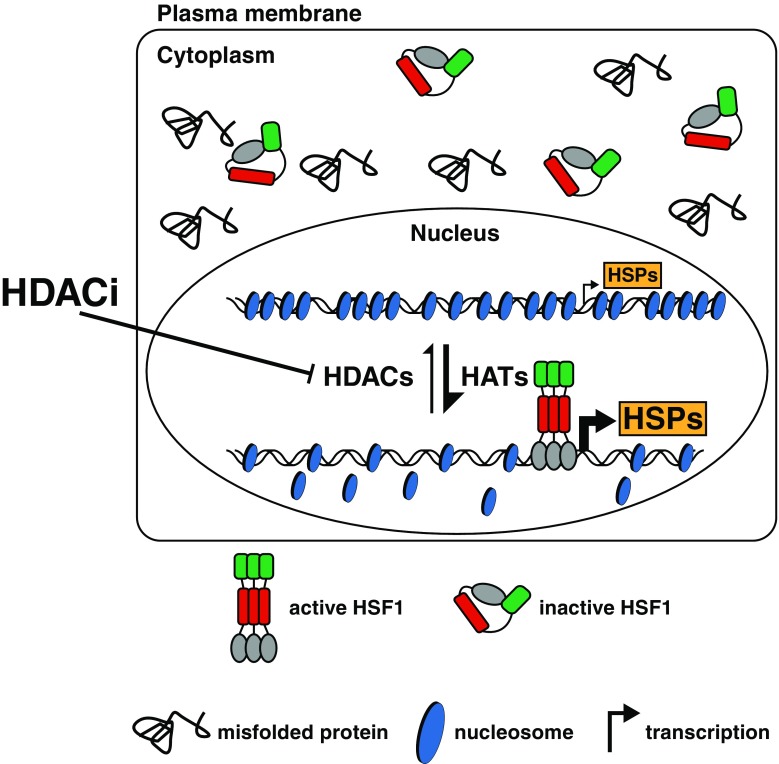
A model for chaperone co-inducing effect of HDAC inhibitors on the heat shock response (HSR). Certain pathological conditions are associated with accumulation of misfolded proteins in cells without inducible expression of Hsps. Inhibition of HDACs increases chromatin accessibility and lowers the threshold for HSF1 activation, thereby enabling and enhancing the HSR. *HATs* histone acetyltransferases, *HDACs* histone deacetylases, *HDACi* HDAC inhibitors, *HSPs* heat shock proteins

Under normal conditions, the expression of Hsps is regulated in an HSF1-independent manner (McMillan et al. 1998; Mahat et al. 2016). Upon proteotoxic stress, HSF1 is activated to bind to its target promoters, which initiates a cascade of events, leading to a release of the paused RNA polymerase II and enabling inducible gene expression (Lis and Wu 1993). Chromatin accessibility at the target promoters of HSF1 influences its ability to recognize and bind DNA (Labbadia and Morimoto 2015; Leach et al. 2016). In all eukaryotes, chromatin accessibility depends on nucleosome occupancy and positioning, controlled by a myriad of histone tail modifications, such as acetylation and methylation (Tessarz and Kouzarides 2014). Acetylation of histones is dynamically regulated by histone acetyltransferases (HATs), enhancing recruitment of the chromatin remodelling complexes, e.g. SWI/SNF, which facilitates the removal of nucleosomes, thereby increasing chromatin accessibility for transcription factors (Tessarz and Kouzarides 2014). Since HDACs counteract acetylation of histones, they maintain compact chromatin conformation and transcriptional silencing. Inhibition of HDACs leads to hyperacetylation of histones followed by their removal from chromatin. Based on our findings that BGP-15 acts as an HDAC inhibitor and increases chromatin accessibility, we propose a model where HDAC inhibition results in enhanced HSR in the event of proteotoxic insults (Fig. 5). Importantly, increased chromatin accessibility lowers the threshold for HSF1 activation and accelerates HSF1 binding to its target promoters. This capacity of lowering the threshold can be used to activate the HSR under those conditions when it is not normally activated (Fig. 1e). Interestingly, the Hsp co-inducing effect of BGP-15 was detected already at 15 min of heat stress for *DNAJB1* mRNA (Fig. 1d), whereas for *HSPA1A/B* mRNA at 30 min of stress. This observation raises the question how these genes respond differently to BGP-15 treatment. A recent study in MEFs showed that the *DNAJB1* promoter is already occupied by HSF1 under control conditions whereas the *HSPA1A* and *HSPA1B* promoters are not (Mahat et al. 2016). Thus, as the *DNAJB1* promoter is occupied by HSF1 before stress, treatment with BGP-15 could increase the pre-existence binding of HSF1 in that specific *locus*, which would not be the case for *HSPA1A* and *HSPA1B* promoters. Alternatively, the difference in the kinetics of inducible expression of HSPA1A/B and DNAJB1 could provide an explanation for observed phenomena. *HSPA1A/B* are unique genes with rapid activation kinetics (Zobeck et al. 2010), which result in a much higher increase of mRNA upon stress when compared with *DNAJB1* (20-fold vs. 0.5-fold) as shown by our results. Assuming that the transcription of *HSPA1A/B* is already functioning close to the maximal rate during the initial phase of HSR, the beneficial effect of BGP-15 would take a longer time to occur.

It is also worth noticing that enhanced expression of Hsps due to combined heat stress and HDAC inhibition was already shown a decade ago in model organisms *Xenopus* (Ovakim and Heikkila 2003) and *Drosophila* (Zhao et al. 2006). Moreover, increased Hsp levels were found in rodents treated with HDAC inhibitors and exposed to various neurological stresses, such as ischaemia (Faraco et al. 2006; Xuan et al. 2012), haemorrhage (Sinn et al. 2007), spinal cord injury (Lv et al. 2011), and middle cerebral artery occlusion (Sinn et al. 2007). However, the mechanism underlying the enhanced HSR by HDAC inhibition has not previously been reported.

HDAC inhibitors belong to a large and diverse family of compounds with beneficial effects in a wide range of animal disease models, including glutamate excitotoxicity (Marinova et al. 2009), chronic pain (Wang et al. 2016), and atrial fibrillation (Lkhagva et al. 2016). Furthermore, chromatin modifiers and especially HDAC inhibitors are currently used as drugs for human diseases, such as cancer (Roche and Bertrand 2016), diabetes (Arguelles et al. 2016), and neurological disorders (Falkenberg and Johnstone 2014). Our finding that BGP-15 acts as an HDAC inhibitor and that HDAC inhibition, in general, enhances the HSR presents a new approach to restore protein homeostasis in protein folding diseases.

## Electronic supplementary material

ESM 1 (DOC 39 kb)
