# Supplementary material for: Chaperone co-inducer BGP-15 inhibits histone deacetylases and enhances the heat shock response through increased chromatin accessibility
Source: Cell Stress Chaperones. 2017 May 4;22(5):717–28. doi: 10.1007/s12192-017-0798-5 (PMC5573690; doi:10.1007/s12192-017-0798-5)
Supplement: Supplementary file 1 — (DOC 39 kb) [file 12192_2017_798_MOESM1_ESM.doc]

**Chaperone Co-inducer BGP-15 Inhibits Histone Deacetylases and Enhances the Heat Shock Response through Increased Chromatin Accessibility**

Marek A. Budzyński^1,2^,*, Tim Crul^3^,*, Samu V. Himanen^1,2^, Noemi Toth^3^, Ferenc Otvos^3^, Lea Sistonen^1,2,$^, Laszlo Vigh^3,$^

^1^ Faculty of Science and Engineering, Cell Biology, Åbo Akademi University, Turku, 20520, Finland

^2^ Turku Centre for Biotechnology, Åbo Akademi University and University of Turku, Turku, 20520, Finland

^3^ Institute of Biochemistry, Biological Research Centre of the Hungarian Academy of Sciences, Szeged, H-6726, Hungary

* These authors contributed equally to this study.

^$^ Corresponding authors: Lea Sistonen Tel: +358 2 215 3311; Fax: +358 2 251 7013; email: lea.sistonen@abo.fi and Laszlo Vigh Tel: +36 62 599 600; Fax: +36 62 432 048; email: vigh@brc.hu

Running title: Enhancing the Heat Shock Response by Chromatin Opening

Keywords: chromatin immunoprecipitation (ChiP), Heat shock factor protein 1 (HSF1), histone deacetylase (HDAC), stress response, transcription, TSA, VPA

Supplemental material:

Supplemental Table S1

Supplemental Table S2

**Supplemental Table S1. Primers and probes used in qRT-PCR experiments.**

Forward primer (F), reverse primer (R), probe (P), dark quencher dye (BHQ). DNAJB1 amplification was done using SYBR green method.

| **Gene** | Primer and probe sequences |
| --- | --- |
| *RNA18S5* | F: 5’-GCAATTATTCCCCATGAACG-3’  R: 5’-GGGACTTAATCAACGCAAGC-3’  P: 5’-FAM-TTCCCAGTAAGTGCGGGTC-BHQ-3’ |
| *HSPA1A/B* | F: 5’-AGGTGCTGGACAAGTGCCAG-3’  R: 5’-AACTCCTCCTTGTCGGCCA-3’  P: 5’-FAM-CATCTCCTGGCTGGACTCCAACACG-BHQ-3’ |
| *DNAJB1* | F: 5’-CTATGGAGAGGAAGGCCTGAA-3’  R: 5’-GGGTCTCCGTGGAATGTGTA-3’ |

**Supplemental Table S2. Primers used in ChIP and MNase experiments**

Forward primer (F), reverse primer (R).

| Gene | Amplicon name | Amplicon range relative to TSS of the gene | Primer sequences |
| --- | --- | --- | --- |
| *HSPA1A* | -1100 | From -1142 to -1070 | F: 5’-TAGTAGGCGGGCCCCAACACC-3’  R: 5’-GCATCTTGCCTCACAGTGCCC-3’ |
|  | -150 | From -241 to -57 | F: 5’-CCCCACACCCTCCCCCTCAG-3’  R: 5’-CTGGGCCAATCAGCGAGCCG-3’ |
|  | TSS | From -77 to +13 | F: 5’-CGGCTCGCTGATTGGCCCAG-3’  R: 5’-GTCAGCGTCTGGTGCCCTGC-3’ |
|  | +1100 | From +1048 to +1147 | F: 5’-CGCTGTCGTCCAGCACCCAG-3’  R: 5’-ACAGCTCTTCGAACCGCGCC-3’ |
| *Notch 4* | -900 | From -967 to -842 | F: 5’-CCCCAGCATCCTTTGTGGAG-3’  R: 5’-GCCTCCAGACTCGTGGTAAA-3’ |
|  | TSS | From -66 to +48 | F: 5’-ACACACACACCAACCTCTCG-3’  R: 5’-GCAGGCTCAGGAGGAAGAAG-3’ |
|  | +500 | From +449 to +523 | F: 5’-GCCAGCTGGTTCTGAAGTAAG-3’  R: 5’-GACTGCCTGATATGGGGAAGAG-3’ |
| *Daxx* | TSS | From -32 to +70 | F: 5’-CTTCCGGCTCTAAGCGGCCTG-3’  R: 5’-TCTGTTGTGGGGTCTGCGGT-3’ |
|  | +1100 | From +1058 to +1163 | F: 5’-TGCTCAACCAGGGCCCTCCAA-3’  R: 5’-TCCGAGCCCCGTGTGGATGG-3’ |
